# Commentary: Absence of behavioral harm following non-efficacious sexual orientation change efforts: A retrospective study of United States sexual minority adults, 2016–2018

**DOI:** 10.3389/fpsyg.2022.997513

**Published:** 2022-10-12

**Authors:** Ilan H. Meyer, John R. Blosnich

**Affiliations:** ^1^School of Law, University of California, Los Angeles, Los Angeles, CA, United States; ^2^Suzanne Dworak-Peck School of Social Work, University of Southern California, Los Angeles, CA, United States

**Keywords:** research methodology, sexual minorities, sexual orientation change efforts (SOCE), suicide, conversion therapy

## Introduction

Using data from *Generations*—a study of a nationally representative sample of sexual minority adults—Blosnich et al. (2020) found that exposure to sexual orientation change effort (SOCE or “conversion therapy”) was associated with twice the odds of lifetime suicidal ideation, 75% increased odds of planning to attempt suicide, and 88% increased odds of a suicide attempt with minor injury compared with people with no history of being exposed to SOCE. Sullins (2022) raised several critiques of that study, including that it did not time the occurrence of SOCE in relation to suicidality, making it difficult to determine the causality, and that it did not sufficiently control for potential confounders of suicidality. Using the *Generations* data, Sullins attempted to address these limitations, but his solutions are flawed, rendering his conclusions invalid.

## Methodological concerns in Sullins' analyses

On the question of timing, Sullins argued that Blosnich et al. (2020) analyses, which relied on the association of lifetime exposure with SOCE and lifetime suicide morbidity, failed to establish whether SOCE exposure preceded suicide morbidity. This is a valid critique, as Blosnich et al. (2020) already said, “To probe causal relationships, future survey items ought to attend to issues of the timing of […] SOCE (e.g., *age of first and last experiences*)” (p. 1029, *emphasis added*). Sullins' purported correction of this limitation uses data on last exposure to SOCE and, relying on this analysis, he incorrectly claimed that his findings demonstrate that SOCE is not associated with suicidal behavior.

Sullins's claim is incorrect because *Generations* data did not indicate the age when a person was first exposed to SOCE—the benchmark for correctly timing suicide morbidity before or after exposure to SOCE. For example, if a respondent indicated that their age of *last* SOCE exposure was 19 years old and their age of suicide attempt was 17 years old, Sullins assumed that the suicide attempt preceded SOCE and, therefore, exonerates SOCE as a potential risk factor. However, it is entirely possible (and as we show below, likely) that the respondent's first SOCE exposure was prior to their suicide attempt at age 17. For example, the person in this example might have started SOCE at age 16 and ended at age 19. If this were the case, Sullins misclassified this person as having had no suicide attempt after their SOCE exposure because of only using the last SOCE exposure for timing. This limitation in the timing of SOCE exposure in the *Generations* data is precisely why we did not use timing in the Blosnich et al. (2020) analysis and defaulted to a lifetime exposure measurement.

Sullins' interpretation of age at last SOCE as his benchmark of exposure is especially concerning because he misrepresented findings on time of SOCE exposure. Sullins claimed that “SOCE therapy is not lifelong or continuous, but is confined to a definite, restricted time in the life course, *usually lasting less than a year*” (p. 2, *emphasis added*). But, in fact, research has shown that most people who were exposed to SOCE had multiple and prolonged exposures. Flentje et al. (2013) found that respondents exposed to SOCE in their study had on average three different episodes of SOCE, lasting from 1 week to 4.5 years per episode. Similarly, Spitzer (2003) reported that the mean duration of SOCE exposure was 4.7 years. Additionally, Salway et al. (2021) found that 30% of their sample of 910 people who experienced SOCE reported *more* than 5 attempts at SOCE. In the *Generations* study, most (73%) of the people who reported a suicide attempt prior to the last exposure to SOCE had experienced SOCE 5 years or less prior to that attempt; that is, their suicide attempt was within the period of a typical SOCE exposure. Thus, using the age of last SOCE exposure cannot indicate whether suicide ideation or attempt indeed preceded SOCE.

Second, Sullins used 1 year (the year prior to the time of the interview) rather than lifetime suicide morbidity as the outcome. But most people experience SOCE at a young age; suicide morbidity, if it occurred, would have been more proximal to the initial SOCE exposure. In the *Generations* sample of adults aged 18–60, SOCE exposure could have happened quite long before the past-year time frame that Sullins uses for his outcome. Among the *Generations'* individuals who reported SOCE exposure, the average age of the *last* SOCE exposure was 18 years, but their average age at the time of the interview was about 33 years. Flentje et al. found that most suicidality reported among people exposed to SOCE occurred *during* the period they were exposed to SOCE. By using past-year suicide morbidity for a risk exposure that for many respondents had occurred decades before, Sullins biased the analysis toward the null. In effect, Sullins argued that because someone is not *currently* suicidal from a SOCE exposure that likely happened 10 or more years earlier, SOCE is not harmful.

On the question of controlling for potential bias due to confounding, it is difficult to assess Sullins' approach as he does not provide a clear conceptual model. He not only includes, among predictors of suicide, a range of stressors but also includes psychological distress. Mood disorders are the strongest predictors of suicidality (Kessler et al., 1999), but psychological distress is misplaced as the control variables in the test of the role of stress on suicide behavior because if SOCE impacted suicide behavior, as we hypothesized, it would work by increasing psychological distress. Controlling for distress, as Sullins does, overcontrols the tested hypothesis. When it comes to choosing control variables, a clearly articulated causal conceptual model might better clarify which variables ought to be included in the statistical model Sullins used. Controlling for a variable that is clearly in the hypothesized causal chain biases results toward the null.

## Conclusion

Sullins' approach to addressing the original study's limitations is built on problematic timing data, the use of a 1 year suicide attempt as an outcome that is far removed from the potential exposure to SOCE, and the overcontrol in his causal models. We, therefore, conclude that Sullins' findings are misleading and his conclusions about the risk posed by SOCE are not valid.

## Author contributions

Both authors contributed equally to the writing of this commentary. All authors contributed to the article and approved the submitted version.

## Funding

The Generations study was funded by the Eunice Kennedy Shriver National Institute of Child Health and Human Development (grant HD078526).

## Conflict of interest

The authors declare that the research was conducted in the absence of any commercial or financial relationships that could be construed as a potential conflict of interest.

## Publisher's note

All claims expressed in this article are solely those of the authors and do not necessarily represent those of their affiliated organizations, or those of the publisher, the editors and the reviewers. Any product that may be evaluated in this article, or claim that may be made by its manufacturer, is not guaranteed or endorsed by the publisher.
